# Supplementary figures and images for: Clonorchis sinensis infection modulates key cytokines for essential immune response impacted by sex
Source: PLoS Negl Trop Dis. 2022 Sep 9;16(9):e0010726. doi: 10.1371/journal.pntd.0010726 (PMC9462580; doi:10.1371/journal.pntd.0010726)

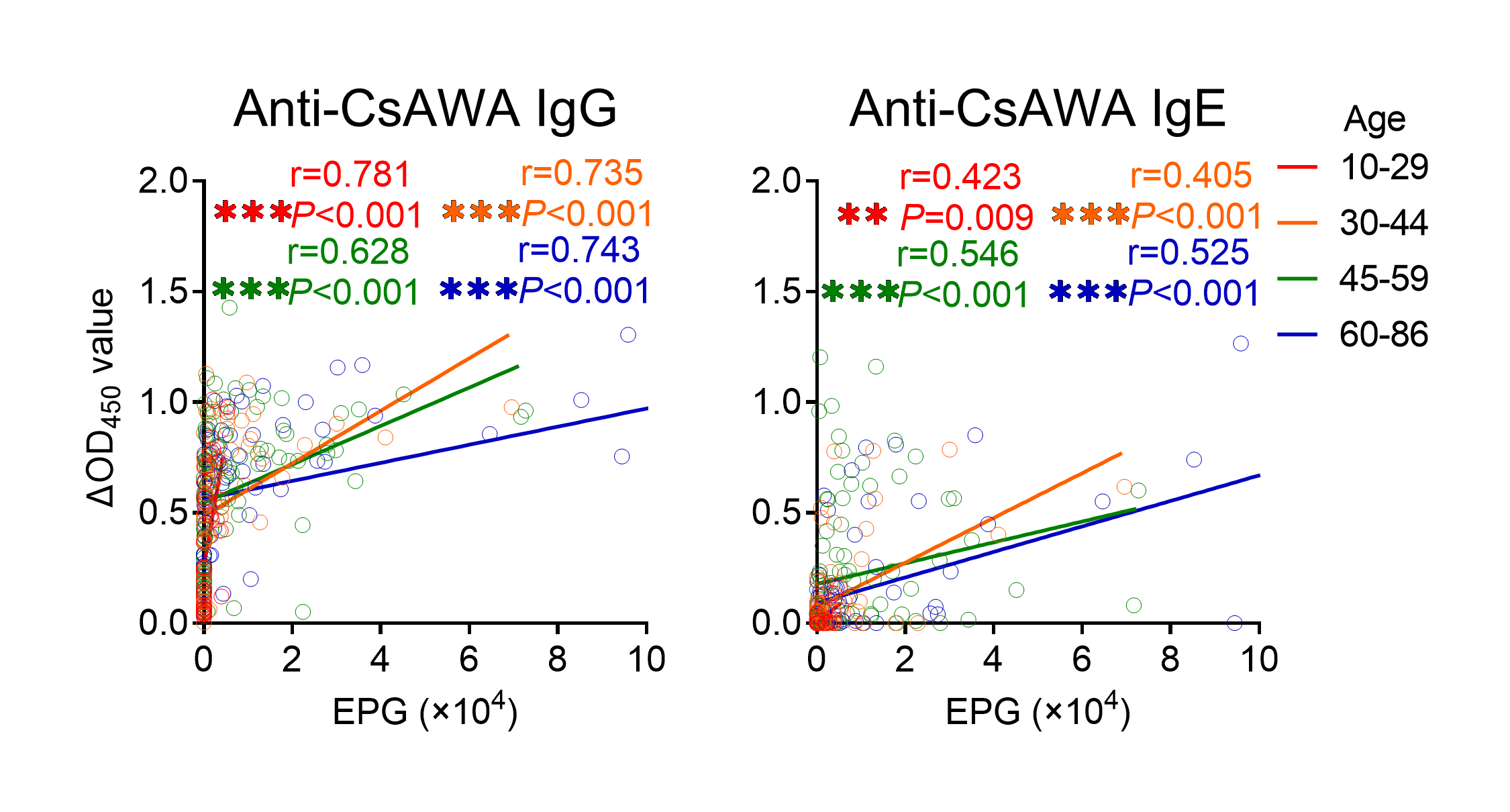

Supplement: S1 Fig — Spearman’s correlation analysis between EPG and serum levels of anti-CsAWA IgG and IgE (n = 37 in 10–29 years, n = 73 in 30–44 years, n = 97 in 45–59 years, and n = 82 in 60–86 years). CsAWA: C. sinensis adult worm antigen, EPG: eggs per gram of feces. The data were shown as the mean ± s.e.m., **P< 0.01, ***P < 0.001, Spearman’s correlation test. (TIF) [file pntd.0010726.s002.tif]

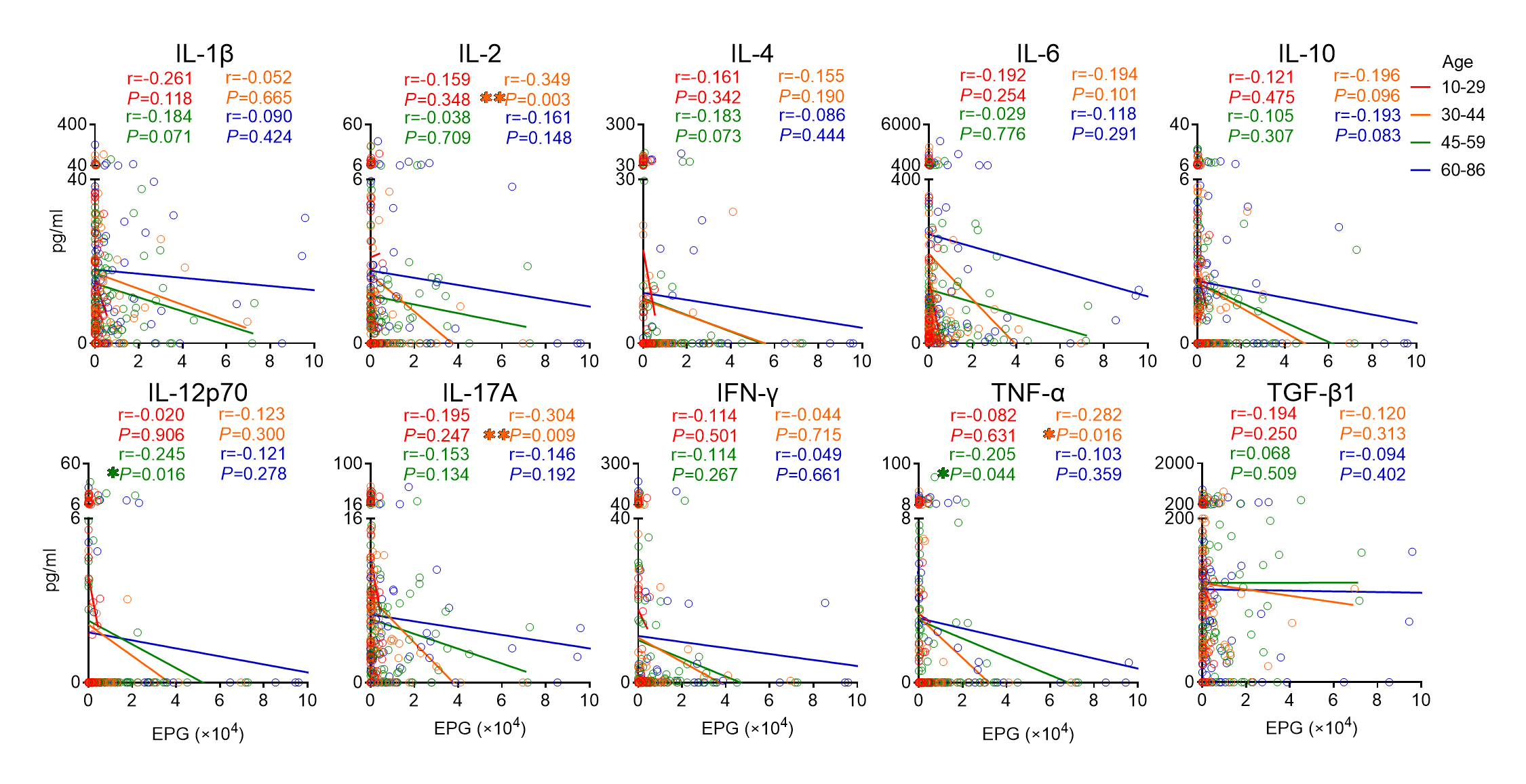

Supplement: S2 Fig — Spearman’s correlation analysis between EPG and serum levels of IL-1β, IL-2, IL-4, IL-6, IL-10, IL-12p70, IL-17A, IFN-γ, TNF-α and TGF-β1 in different age groups (n = 37 in 10–29 years, n = 73 in 30–44 years, n = 97 in 45–59 years, and n = 82 in 60–86 years). CsAWA: C. sinensis adult worm antigen, EPG: eggs per gram of feces. The data were shown as the mean ± s.e.m., *P< 0.05, **P< 0.01, Spearman’s correlation test. (TIF) [file pntd.0010726.s003.tif]

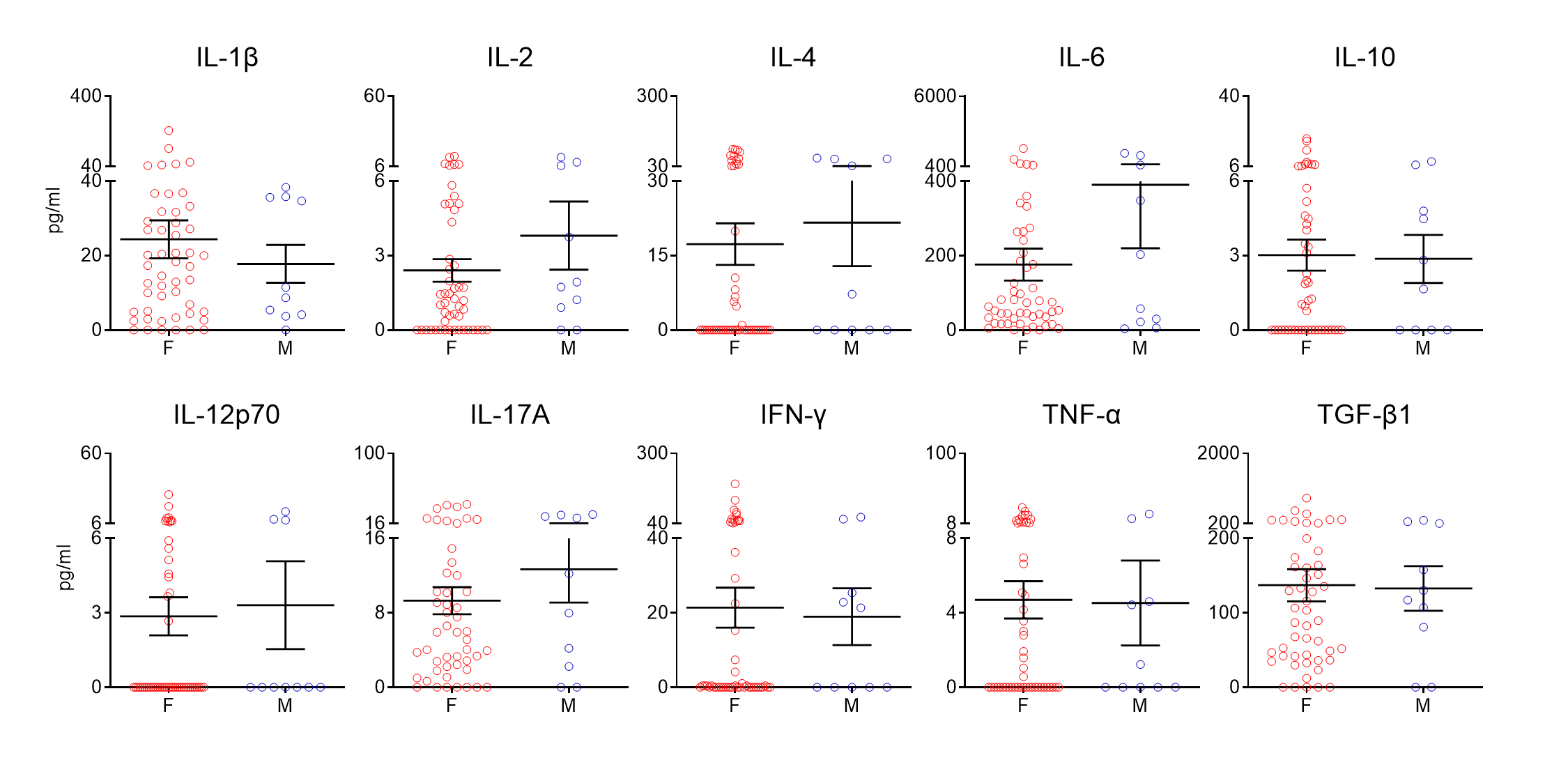

Supplement: S3 Fig — Serum levels of IL-1β, IL-2, IL-4, IL-6, IL-10, IL-12p70, IL-17A, IFN-γ, TNF-α and TGF-β1 in Cs- individuals (n = 50 in female and n = 10 in male). Cs: C. sinensis infection, F: female, M: male. The data were shown as the mean ± s.e.m., Mann-Whitney U test. (TIF) [file pntd.0010726.s004.tif]

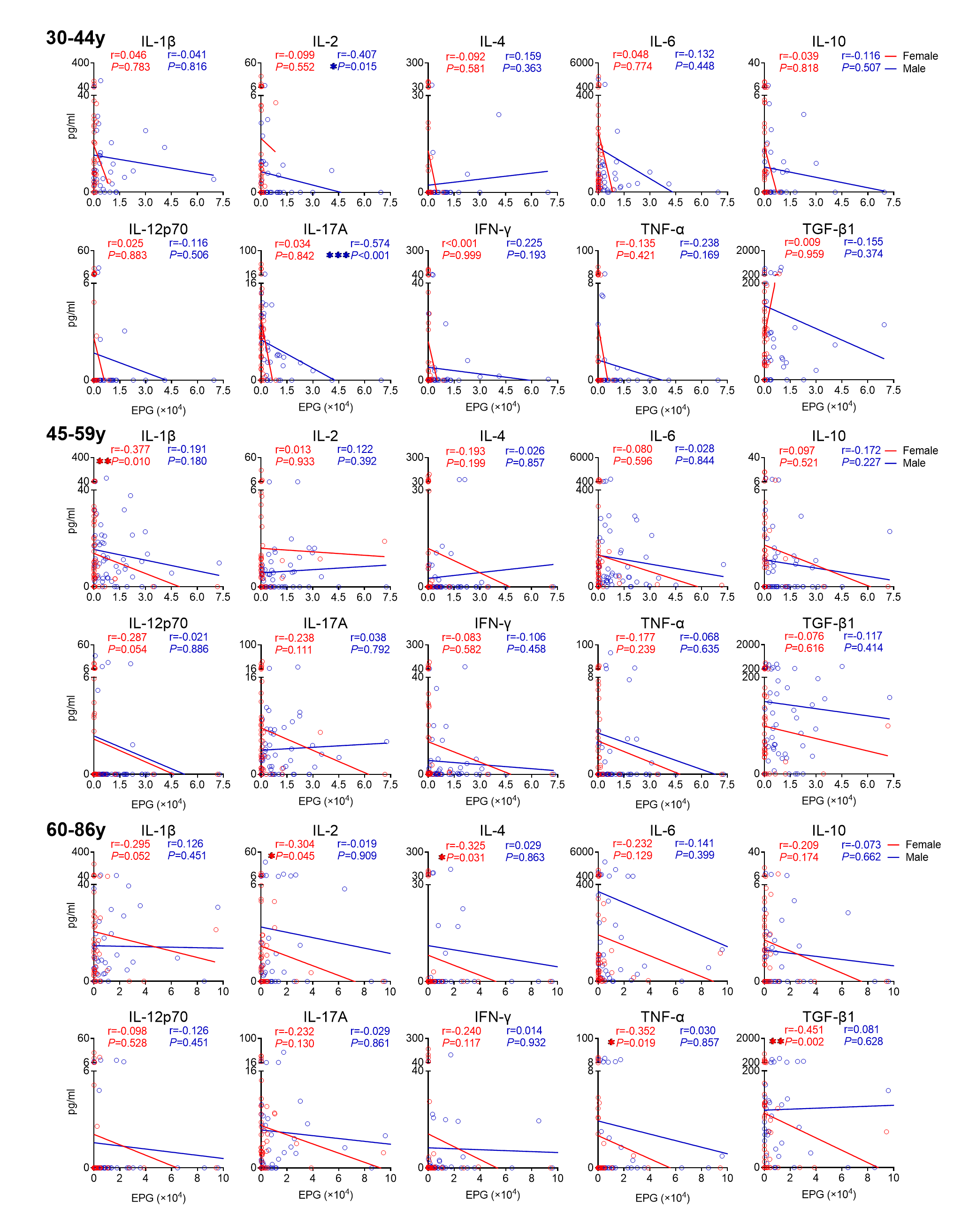

Supplement: S4 Fig — Spearman’s correlation analysis between EPG and serum levels of IL-1β, IL-2, IL-4, IL-6, IL-10, IL-12p70, IL-17A, IFN-γ, TNF-α and TGF-β1 in 30–44 y (n = 38 in female and n = 35 in male), 45–59 y (n = 46 in female and n = 51 in male) and 60–86 y (n = 44 in female and n = 38 in male). EPG: eggs per gram of feces. The data were shown as the mean ± s.e.m., *P< 0.05, **P< 0.01, Spearman’s correlation test. (TIF) [file pntd.0010726.s005.tif]

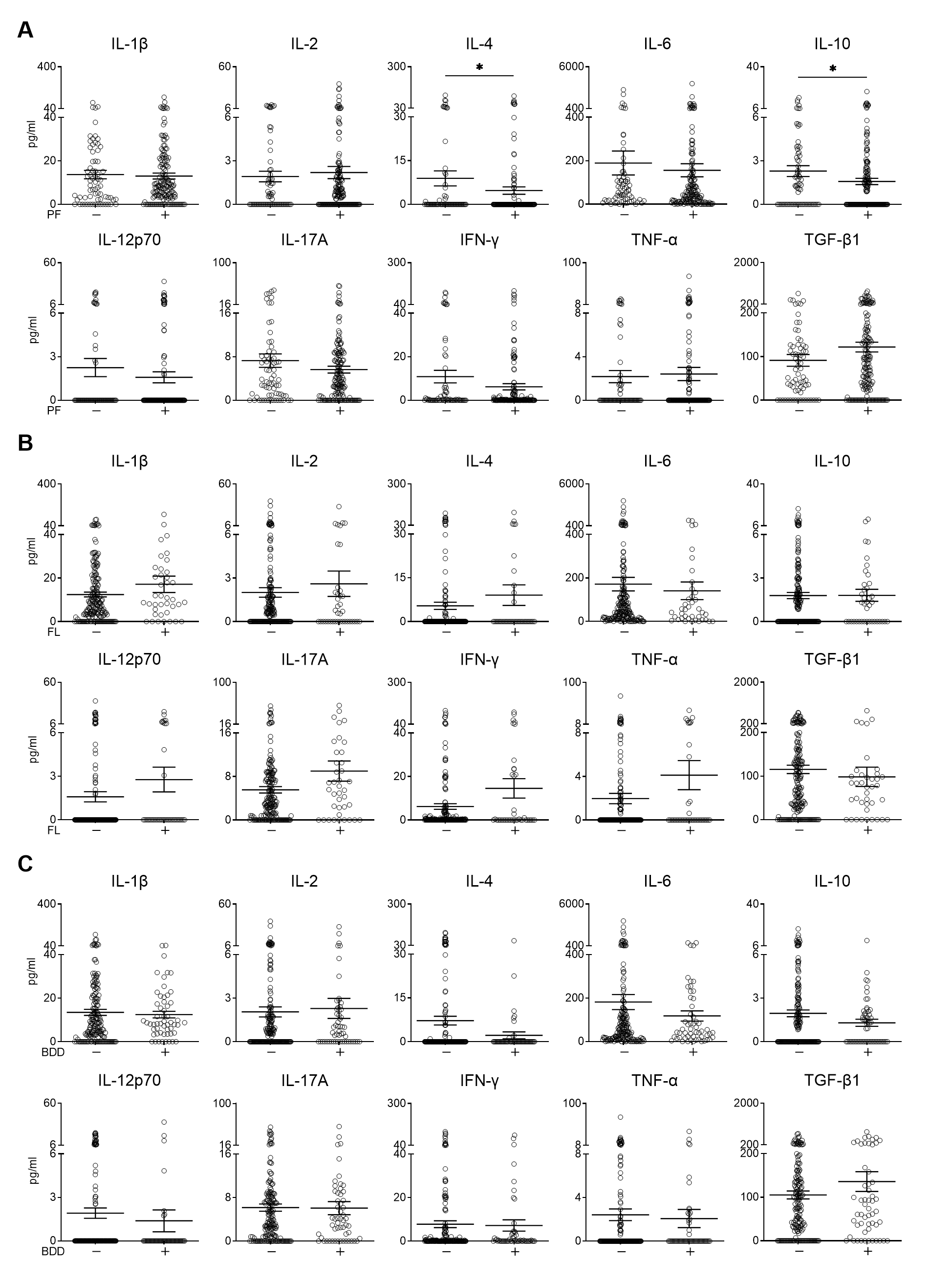

Supplement: S5 Fig — (A) Serum levels of IL-1β, IL-2, IL-4, IL-6, IL-10, IL-12p70, IL-17A, IFN-γ, TNF-α and TGF-β1 in Cs+ individuals with (n = 159) or without periductal fibrosis (n = 70). (B) Serum levels of cytokines in Cs+ individuals with (n = 39) or without fatty liver (n = 190). (C) Serum levels of cytokines in Cs+ individuals with (n = 55) or without bile duct dilatation (n = 174). PF: periductal fibrosis, FL: fatty liver, BDD: bile duct dilatation. The data were shown as the mean ± s.e.m., *p < 0.05, Mann-Whitney U test. (TIF) [file pntd.0010726.s006.tif]
