# Supplementary material for: Clonorchis sinensis infection modulates key cytokines for essential immune response impacted by sex
Source: PLoS Negl Trop Dis. 2022 Sep 9;16(9):e0010726. doi: 10.1371/journal.pntd.0010726 (PMC9462580; doi:10.1371/journal.pntd.0010726)
Supplement: Attachment — Submitted filename: Response to PNTD Reviewers.pdf [file pntd.0010726.s007.pdf]

- **Response to Reviewers**

We thank all 3 reviewers for their critical evaluation of this manuscript. All comments are valuable and very helpful for revising and improving our manuscript, as well as the important guiding significance to our researches. In the revised manuscript, we have addressed the reviewers' concerns thoroughly. Below are our point-by-point responses to the reviewers' comments (shown in blue). The changes made to the text of the revised manuscript are highlighted in yellow. Page numbers corresponding to these changes are provided in this response letter.

Reviewer #1:

Summary and General Comments:
In this study, the authors found the characteristics of host immune response of human infected with *C. sinensis*. After *C. sinensis* infection, antigen-specific IgG and IgE levels were higher than control people. In addition, the antigen-specific IgE level had sex difference. *C. sinensis* infection caused diminished Th1 cytokines, Th2 cytokine, and the changes of cytokine milieus also had sex difference, indicating that the immune response was suppressed and had sexual dimorphism. However, there was no significant correlation between the dampened cytokine profiles and the hepatobiliary morbidities. This study provides a better understanding of how worms affect immune responses and cause a long-term immune alternation in humans with *C. sinensis* infection.

Response: We thank the reviewer for carefully reading our manuscript and giving constructive comments.

However, there are some problems in the article. The major comments are as follows:

1. The specific IgE level and cytokine milieus had sex differences in *C. sinensis* infection. PCA analysis indicated that there was a relation between cytokine and antibodies clusters in male and female individuals infected with *C. sinensis*. Could you explain the factors resulted in the sex difference of cytokine milieus?

Response: Both genetic and hormonal factors may result in the sex difference of cytokine milieus [1, 2]. For example, genes on the X chromosome code for numerous proteins involved in immune processes, including pattern recognition receptors (PRRs, e.g., *TLR7* and *TLR8*), transcriptional factors (e.g., *FOXP3*) and main members in nuclear factor-κB pathway (e.g., *IRAK-1*and *NEMO*), which are important in immune cell activation and cytokine production [3, 4]. Sex hormones can influence the function of host immune cells by binding to specific receptors that are expressed in most immune cells, such as lymphocytes, macrophages and dendritic cells [1]. Moreover, hormone response elements are present in the promoters of several immune genes, thus sex hormones may directly alter gene expression and immune response [2]. It was reported that the expression of PRRs (e.g., TLR4 and TLR9) could be regulated by sex hormone[5]. Innate immune cells from males express higher levels of TLR4

and produce more pro-inflammatory cytokine TNFα and chemokine CXCL10 than female cells both constitutively and following activation [1, 6]. Activation of TLR9 in PBMCs from human males results in more IL-10 production compared with cells from females, which is positively correlated with androgen concentration in males [1].
This discussion is added to the revised manuscript on Page 17 and 18.

2. Perhaps sex hormones related to the sex differences of specific IgE level and cytokine milieus. The detection of sex hormones was suggested.

Response: Sex hormones modulate both humoral and cell-mediated immune pathway. Many studies showed clinical differences between men and women in terms of inflammatory markers can be explained by their levels of sex steroids [7, 8]. In this study, we found that female individuals developed less specific IgE and had more reduction of systemic cytokines compared to males with similar infection intensity. We focused on the immune profiling and reported a new characteristic of host immune response in clonorchiasis. Due to the problem of insufficient specimen, we have not analyzed sex hormone levels. To further explore the underlying mechanisms of immune suppression in clonorchiasis, the roles of sex hormones are worth to be investigated.
The information has been added in the Discussion on Page18.

3. In the discussion, line 279: Wang and colleagues carried out an experimental model in rats and found that immune response presented a tendency to Th2 type by expressing transient high levels of IgG, IgE and IL-4. In this study, Th2 cytokine (IL-4) reduced markedly in *C. sinensis* infected people. In the discussion, the low level of IL-4 might relate to the immune suppression caused by long time duration of infection. It is interesting to explore the time of IL-4 level alternation.

Response: We agree with the reviewer that it is interesting to explore the time of IL-4 level alternation. This is an important question that we are actively studying. As mentioned in ethics statement, individuals with *C. sinensis* infection were treated in time, so it is hard to follow IL-4 level alternation in patients with current infection. According to literatures, in *C.sinensis* infected mice, IL-4 production by splenocytes increased (> threefold) until 2-4 weeks post-infection, but declined thereafter [9]. In rat models, compared with control, IFN-γ and IL-4 levels were elevated post infection, and both decreased to lower levels at week 16 after primary infection [10]. In human beings, *C. sinensis* usually causes long-term infection. Untreated, infection may persist for up to 25–30 years[11]. With long-standing chronic infection, immune suppression might be the dominant phenotype in human clonorchiasis. It is interesting to establish a long-standing, persistent infection model (e.g., more than 24 weeks) to track the dynamic changes of cytokines and investigate the intrinsic mechanism.
The information has been added in the Discussion on Page15 and 16.

4. In the part of “abstract”, please illustrate the numbers of individuals infected with or without *Clonorchis sinensis* respectively.

.
Response: As suggested, the information has been added on Page 2.

5. There are some mistakes in grammar, please revise them, such as line 69: there is a "," after meanwhile and please revise it to "meanwhile,"; line 87: "because the carcinogenesis in fatal cholangiocarcinoma" turn to "because of the carcinogenesis in fatal cholangiocarcinoma".

Response: We apologize for the errors and have corrected them in the revised manuscript. We thank the reviewer for carefully reading our manuscript again.

Reviewer #2:

Methods:
The status or level of immune response may be related to factors such as gender and duration of infection, as the author mentioned in the manuscript. In fact, the age of the patient was the factor most likely associated with the duration of the infection. Therefore, it is suggested that the authors stratified the data collected based on gender and age. Otherwise, some valuable information or features may be annihilated. If the stratified analysis is not possible due to the number of cases, the author should also discuss this issue appropriately.

Response:
As suggested, we analyzed the levels of specific antibodies and key cytokines for essential immune response in different age groups (10-29 years, 30-44 years, 45-59 years, and 60-86 years). Elevated levels of serum IgG and IgE were positively correlated with the intensity of infection (EPG) in all four age groups (new Supplementary Figure 1). Cytokines showed decreasing trend with increasing EPG in all age groups (new Supplementary Figure 2).
We also compared the cytokine levels between females and males in different age groups. In general, both females and males had suppressed cytokine milieus post *C. sinensis* infection in 30-44 years, 45-59 years, and 60-86 years groups. Female individuals showed more reduction in cytokines than male individuals (new Supplementary Figure 4). Since there are only 4 female *Cs*+ cases in 10-29 years group, this group data has not been shown.
These requested new data are discussed on Page 9, 10 and 11.

Conclusions:
This study was mainly based on serological data and did not study the mechanism of host immunosuppression caused by *C. sinensis* infection. Therefore, the expression "This finding expends our understanding of how worms affect immune response" in the abstract and the conclusion of the manuscript is inadequate.

Response: The sentence in question has been revised. The words in Summary and General Comments given by Reviewer #1 "This study provides a better understanding of how

worms affect immune responses and cause a long-term immune alternation in humans with *C. sinensis* infection." were used in the abstract and revised manuscript on Page 2 and Page 18.

Summary and General Comments:
In this study, the immune response status of clonorchiasis individuals and uninfected persons was observed and analyzed. The main findings are: the serum specific IgE level of female infected patients was lower than that of male infected patients, and Th1, Th2 and Th17 cytokines were lower and more obvious in females. There was no correlation between cytokine levels and hepatobiliary morbidity. This study has certain significance for further understanding the immunological characteristics of clonorchiasis and the mechanism of worm infection regulating host immune response. The English writing is clear and fluent.

Response: We thank the reviewer for positive and insightful comments.

However, there are also some obvious deficiencies, and it is suggested that the author make some modifications. Besides, Line 110: the sentence "The eggs per gram of feces (EPG) is calculated by the average number of eggs in three Kato-Katz smears by a factor of 24." Is not correct.

Response: The sentence questioned has been revised as follows:
The eggs per gram of feces (EPG) is calculated by multiplying the average of three Kato-Katz smears with a factor of 24. (Page 6).

Reviewer #3:

Summary and General Comments:
This manuscript showed us an interesting story that *C. sinensis* infection is strongly related to the immune suppression in human and declared that sex differences can shape the immune milieus of clonorchiasis. These results are some interesting.

Response: We appreciated the reviewer for her/his critical evaluation of this manuscript.

However, we have some comments.

1. The author showed that phenotypes were significantly in female than those in males. However, for detection the sex difference, the results are very limited. Sex difference is better considered the hormonal difference from the different age. Whether there exists some difference at baseline levels from the male and female should be illustrated. And I think if the author can add some animals experiment evidence can be better.

Response: As suggested, we analyzed the levels of specific antibodies and key cytokines for essential immune response in different age groups (10-29 years, 30-44 years, 45-59 years,

and 60-86 years). Elevated levels of serum IgG and IgE were positively correlated with the intensity of infection (EPG) in all four age groups (new Supplementary Figure 1). In addition, cytokines showed decreasing trend with increasing EPG in all age groups (new Supplementary Figure 2).

The cytokine levels in females and males without *C. sinensis* infection were also compared. No significant difference was observed. The baseline levels of cytokines were comparable in females and males (Supplementary Figure 3).

We further compared the cytokine levels between females and males in different age groups. In general, both females and males had suppressed cytokine milieus post *C. sinensis* infection in 30-44 years, 45-59 years, and 60-86 years groups. Female individuals showed more reduction in cytokines than male individuals (new Supplementary Figure 4). Since there are only 4 female *Cs*+ cases in 10-29 years group, this group data have not been shown.

We agree with the reviewer that animal experiments are important to explore the mechanism of host immunosuppression caused by *C. sinensis* infection. According to literatures, in *C. sinensis* infected mice, IL-4 production by splenocytes increased (> threefold) until 2-4 weeks post-infection, but declined thereafter [9]. In rat models, compared with control, IFN-γ and IL-4 levels were elevated post infection, and both decreased to lower levels at week 16 after primary infection [10]. In human beings, *C. sinensis* usually causes long-term infection. Untreated, infection may persist for up to 25–30 years [11]. With long-standing chronic infection, immune suppression might be the dominant phenotype in human clonorchiasis. It is interesting to establish a long-standing, persistent infection model (more than 24 weeks) to track the dynamic changes of cytokines and investigate the intrinsic mechanism. This is an important question that we are actively studying. In this study, we mainly focused on the immune profiling in clonorchiasis and reported a new immune characteristic in this disease.

The information has been added on Page 9, 10, 11 and in the Discussion on Page 15 and 16.

2. For the cases included in the experiment, it need described whether these patients have any other diseases or co-infection.

Response: As suggested, we collected the information about whether participants have co-infection with soil-transmitted helminths (new **Supplementary Table**). Excluding co-infection with soil-transmitted helminths, individuals with *C. sinensis* still had significantly higher antigen-specific IgG and IgE levels, and diminished serum levels of IL-1β, IL-2, IL-4, IL-12p70, IL-17A, IFN-γ and TNF-α (new **Figure 7**). This new data are discussed on Page 13.

3. Colors of the pictures had better have consistent.

Response: As suggested, we checked all colors of our pictures. Colors of original Supplementary Figure 1 (Supplementary Figure 5 in the revised manuscript) have been changed.

Reference:

1. Klein SL, Flanagan KL. Sex differences in immune responses. Nature reviews Immunology. 2016;16(10):626-38. Epub 2016/08/23. doi: 10.1038/nri.2016.90. PubMed PMID: 27546235.
2. vom Steeg LG, Klein SL. SeXX Matters in Infectious Disease Pathogenesis. PLoS pathogens. 2016;12(2):e1005374. Epub 2016/02/20. doi: 10.1371/journal.ppat.1005374. PubMed PMID: 26891052; PubMed Central PMCID: PMCPMC4759457.
3. Libert C, Dejager L, Pinheiro I. The X chromosome in immune functions: when a chromosome makes the difference. Nature reviews Immunology. 2010;10(8):594-604. Epub 2010/07/24. doi: 10.1038/nri2815. PubMed PMID: 20651746.
4. Spolarics Z. The X-files of inflammation: cellular mosaicism of X-linked polymorphic genes and the female advantage in the host response to injury and infection. Shock. 2007;27(6):597-604. Epub 2007/05/17. doi: 10.1097/SHK.0b013e31802e40bd. PubMed PMID: 17505297.
5. Rettew JA, Huet-Hudson YM, Marriott I. Testosterone reduces macrophage expression in the mouse of toll-like receptor 4, a trigger for inflammation and innate immunity. Biol Reprod. 2008;78(3):432-7. Epub 2007/11/16. doi: 10.1095/biolreprod.107.063545. PubMed PMID: 18003947.
6. Aomatsu M, Kato T, Kasahara E, Kitagawa S. Gender difference in tumor necrosis factor-alpha production in human neutrophils stimulated by lipopolysaccharide and interferon-gamma. Biochemical and biophysical research communications. 2013;441(1):220-5. Epub 2013/10/22. doi: 10.1016/j.bbrc.2013.10.042. PubMed PMID: 24140406.
7. Lefevre N, Corazza F, Valsamis J, Delbaere A, De Maertelaer V, Duchateau J, et al. The Number of X Chromosomes Influences Inflammatory Cytokine Production Following Toll-Like Receptor Stimulation. Frontiers in immunology. 2019;10:1052. Epub 2019/05/31. doi: 10.3389/fimmu.2019.01052. PubMed PMID: 31143188; PubMed Central PMCID: PMCPMC6521177.
8. Cunningham M, Gilkeson G. Estrogen receptors in immunity and autoimmunity. Clin Rev Allergy Immunol. 2011;40(1):66-73. Epub 2010/03/31. doi: 10.1007/s12016-010-8203-5. PubMed PMID: 20352526.
9. Choi YK, Yoon BI, Won YS, Lee CH, Hyun BH, Kim HC, et al. Cytokine responses in mice infected with Clonorchis sinensis. Parasitology research. 2003;91(2):87-93. Epub 2003/08/05. doi: 10.1007/s00436-003-0934-2. PubMed PMID: 12898229.
10. Wang X, Liang C, Chen W, Fan Y, Hu X, Xu J, et al. Experimental model in rats for study on transmission dynamics and evaluation of Clonorchis sinensis infection immunologically, morphologically, and pathologically. Parasitology research. 2009;106(1):15-21. Epub 2009/09/17. doi: 10.1007/s00436-009-1622-7. PubMed PMID: 19756744.
11. Qian M-B, Utzinger J, Keiser J, Zhou X-N. Clonorchiasis. The Lancet. 2016;387(10020):800-10. doi: 10.1016/s0140-6736(15)60313-0.
